# 17-year follow-up of association between telomere length and all-cause mortality, cardiovascular mortality in individuals with metabolic syndrome: results from the NHANES database prospective cohort study

**DOI:** 10.1186/s13098-023-01206-7

**Published:** 2023-12-02

**Authors:** Lijiao Xiong, Guangyan Yang, Tianting Guo, Zhaohao Zeng, Tingfeng Liao, Yanchun Li, Ying Li, Fujuan Chen, Shu Yang, Lin Kang, Zhen Liang

**Affiliations:** 1 Department of Geriatrics, the Second Clinical Medical College, Jinan University, Shenzhen People’s Hospital, Shenzhen, China; 2 Guangdong Provincial Clinical Research Center for Geriatrics, Shenzhen Clinical Research Center for Geriatrics, Shenzhen People’s Hospital, The Second Clinical Medical College, Jinan University; The First Affiliated Hospital, Southern University of Science and Technology, Shenzhen, China; 3 Department of Orthopedics, Ganzhou Hospital of Guangdong Provincial People’s Hospital, Ganzhou Municipal Hospital, 341000 Ganzhou, Jiangxi province China; 4 Department of Neurology, Shenzhen People’s Hospital, The Second Clinical Medical College, Jinan University; The First Affiliated Hospital, Southern University of Science and Technology, Shenzhen, China; 5 Department of General Practice Medicine, The First Affiliated Hospital of Jinan University, 510630 Guangzhou, China

**Keywords:** Leucocyte telomere length, Metabolic syndrome, All-cause mortality, Cardiovascular Disease mortality, NHANES

## Abstract

**Background:**

The relationship between leukocyte telomere length (LTL) and mortality risk in individuals with metabolic syndrome (MetS) remains poorly understood. This study aimed to investigate the association between telomere length and long-term all-cause mortality, and cardiovascular disease (CVD) mortality, in individuals with MetS in the United States.

**Methods:**

A total of 1980 participants with MetS aged 18 years or older from the National Health and Nutrition Examination Survey (NHANES) prospective cohort study (1999–2002) were included in this cohort study. Medical records review was used to identify the cause of deaths as of December 2018. We employed Kaplan-Meier curves, fitted curves, and Cox proportional hazards regression models to estimate hazard ratios (HRs) for all-cause and CVD mortality, stratified by tertiles of LTL.

**Results:**

Over a median follow-up of 17.75 years of participants with metabolic syndrome, 819 deaths occurred, including 231 cardiovascular deaths. After adjusting for multiple covariates, participants with shorter telomere length had a significantly higher risk of all-cause mortality (HR, 1.33; 95% CI, 1.11–1.6) and CVD mortality (HR, 1.36; 95% CI, 0.96–1.93) compared with those in the highest tertile of telomere length. All-cause mortality (P < 0.001) and cardiovascular disease mortality (P = 0.028) followed a similar pattern across tertiles of telomere length.

**Conclusion:**

In individuals with MetS, shorter telomere length is associated with increased risks of death from cardiovascular disease and all causes. The underlying mechanisms and clinical implications of these findings require additional investigation.

## Introduction

Metabolic syndrome (MetS) is a highly prevalent constellation of metabolic abnormalities including central obesity, hyperglycemia, hypertension, and dyslipidemia [1, 2]. The prevalence of MetS has increased significantly in recent years, and it is estimated that 34.7% of adults in the United States have MetS [3]. It is a significant global health challenge due to its association with increased cardiovascular disease (CVD) and mortality risk [4]. The development of metabolic syndrome is also linked to a higher lifetime risk of CVD and a shorter life expectancy without CVD [5].

Telomere length (TL) has gained attention as a possible biomarker of age and age-related illnesses in recent years. Telomeres are DNA-protein complexes that prevent damage to and malfunction at the ends of chromosomes. Cellular aging is associated with a reduction in leukocyte telomere length (LTL), which can trigger genomic instability, DNA damage, cellular senescence, or apoptosis. Several age-related disorders, such as cardiovascular disease and cancer, have this as a proposed mechanism of onset [6]. Increasing evidence from recent studies suggests that telomere length may contribute to metabolic health [4, 7, 8]. Reduced LTT has been connected to cellular senescence, inflammation, and an increased risk of age-related illnesses like CVD, diabetes, and mortality [6, 9].

Shorter LTL is associated with an increased risk of mortality in contemporary humans, particularly for non-cancer causes of death such as cardiovascular disease, and LTL may serve as a marker for the natural lifespan limit in humans [10]. However, studies have found contradictory results, indicating that longer leukocyte telomere length increases the risk of cardiovascular mortality in patients with type 2 diabetes [11]. Shorter TL is strongly correlated with multiple components of metabolic syndrome, such as abdominal obesity, dyslipidemia, hyperglycemia, and overall metabolic health, and it also serves as a predictor for an unfavorable metabolic profile [12, 13]. Each standard deviation decrease in TL was associated with a 1.19-fold higher odds of having MetS [12]. These associations persist even after a six-year follow-up [12]. Mendelian randomization study revealed a paradoxical association between LTL (leukocyte telomere length) and the risk of metabolic syndrome [14]. It was observed that higher BMI (body mass index) was linked to shorter LTL, while higher levels of low-density lipoprotein cholesterol were associated with longer LTL [14]. Obesity may be linked to shorter LTL through the mechanisms of increased subclinical inflammation and lower circulating levels of linoleic acid [14]. In another Mendelian randomization study, it was found that longer LTL was associated with a higher waist-to-hip ratio adjusted for body mass index, elevated blood pressure, and increased risk of metabolic syndrome [15]. However, there is limited data regarding how LTL relates to mortality risk in those who have MetS. Consequently, the purpose of this investigation was to examine the potential association between LTL and long-term all-cause and CVD mortality risk in MetS patients in the United States.

## Materials and methods

### Data sources and preparation

Information from the prospective cohort research known as the National Health and Nutrition Examination Survey (NHANES) was analyzed for this paper. NHANES is a large-scale, nationally representative survey of citizens residing in the United States who are not in institutional settings. Each participant gave their written informed permission after the study was authorized by the NCHS institutional review board. Using a multilevel, stratified probability design, the survey sampled 5,000 participants every year, who were subjected to standardized questionnaires and physical examination procedures. Since 1999, the survey has been carried out, with updated data sets made accessible every two years at https://www.cdc.gov/nchs/nhanes/index.htm. The institutional review board at Shenzhen People’s Hospital determined that the study did not require informed permission because it used publically available, de-identified data.

The NHANES data utilized in this study were collected between 1999 and 2002 and were made available to the public. Our study was limited by the following criteria for elimination: Missing data on telomere length (n = 13,296), height (n = 161), weight (n = 80); marital status (n = 364), education (n = 8), alcohol consumption (n = 353), tobacco use (n = 8), annual family income (n = 223), chronic kidney disease (n = 32), heart attack (n = 7), congestive heart failure (n = 15), coronary heart disease (n = 22), hypertension (n = 1), angina (n = 1), stroke (n = 4), anemia (n = 2), mortality (n = 3), and Non-Mets (n = 4427). Overall, 1980 individuals with MetS were enrolled in this study.

### Metabolic syndrome

The criteria for diagnosing MetS include the presence of three or more specific abnormalities out of a total of five, as defined by the American Endocrine Association and the American Society of Clinical Endocrinology. These criteria are elevated waist circumference, elevated triglyceride levels, low levels of high-density lipoprotein cholesterol, elevated blood pressure, and elevated fasting plasma glucose levels. Abdominal obesity, measured by an increased waist circumference, is a significant risk factor for MetS. This criterion is gender-specific, with recommended cutoff values of ≥ 88 cm for females and ≥ 102 cm for males. Elevated triglyceride levels, or treatment for hypertriglyceridemia, is another diagnostic criterion for MetS, with a fasting triglyceride level of ≥ 150 mg/dL considered elevated. Low levels of high-density lipoprotein cholesterol (HDL-C), often referred to as “good” cholesterol, is also a MetS diagnostic criterion. The cutoff values differ between genders, with values < 40 mg/dL for males and < 50 mg/dL for females. Elevated blood pressure, particularly systolic blood pressure, is a major risk factor for heart disease, stroke, and kidney disease. A systolic blood pressure of ≥ 130 mmHg or diastolic blood pressure of ≥ 85 mmHg, or both, are considered indicative of MetS. Individuals who take antihypertensive medication are also considered to meet this criterion. Finally, elevated fasting plasma glucose levels or drug-treated hyperglycemia is also a diagnostic criterion for MetS. Fasting plasma glucose levels ≥ 100 mg/dL is considered a sign of impaired glucose tolerance, which can lead to type 2 diabetes [16].

### Telomere length

Blood samples were obtained from the study participants using standardized protocols. The telomere length assay was performed using a polymerase chain reaction. Telomere length was measured as a relative ratio against standard reference DNA (T/S), with each sample tested three times on three different days, in duplicate wells (yielding six data points in total). Detailed information regarding the laboratory methodology, quality control procedures, and data analysis can be found on the NHANES website, under the laboratory section (http://cdc.gov/nchs/nhanes). The interassay coefficient of variation was determined to be 6.5%. Results are presented as the mean T/S ratio along with its corresponding standard deviation. We transformed this to base pairs for analysis based on previous studies [17, 18].

### All-cause and CVD mortality

All-cause mortality and CVD mortality were the primary outcomes of interest in this study. All-cause mortality was defined as the number of participants who died due to any cause after completing their baseline survey but before December 31, 2018. The International Classification of Diseases, Tenth Revision (ICD-10) codes were used to identify causes of death to obtain data on mortality follow-up from NHANES Public-use Linked Mortality Files (https://www.cdc.gov/nchs/data-linkage/mortality.htm). ICD-10 codes are alphanumeric codes that are used to track diseases and other health-related problems [19]. By using these codes, researchers can track and categorize deaths based on the leading causes of death CVD deaths were categorized using certain ICD codes (054–068). These include coronary artery disease, heart failure,ischemic heart diseases, pericardium disease, acute myocarditis, other heart disease and peripheral artery disease. By categorizing deaths due to CVD using specific ICD codes, researchers can accurately track the number of deaths related to cardiovascular disease.

### Covariates

Our study included various clinical and demographic factors as covariates to account for potential sources of confounding. These covariate variables comprised age, sex, body mass index (BMI), race and ethnicity, educational level, marital status, smoking status, alcohol drinking status, annual family income, and chronic diseases. The relevant information on these covariates was obtained from survey responses in NHANES. Participants were categorized according to five racial/ethnic groups: Mexican American, other Hispanic, non-Hispanic White, Black, or Other (including multiracial). Less than high school, high school graduate or equivalent, Some College or AA degree, and college graduate or above were the educational classifications [20]. Marital status was described using seven categories as follows: Never married, Married, Widowed, or Divorced or Separated. Medical conditions such as anemia, angina, heart attack, congestive heart failure, coronary heart disease, chronic kidney disease, asthma, chronic obstructive pulmonary disease (COPD), diabetes mellitus, hypertension, hyperlipidemia, and stroke were diagnosed by a physician or other qualified healthcare professional. The smoking and drinking behaviors were grouped into three categories: never, past, and current use. Using the standard formula, BMI was calculated as weight (kg)/[height (m^2^) × height (m^2^)].

### Statistical analysis

For continuous variables, 95% confidence intervals (CIs) were provided, while for categorical variables, percentage frequencies were provided. T-tests and χ2 tests were used to compare continuous and categorical data. No imputation approach was applied because all variables had low missing data rates. The mortality risk is calculated using Cox proportional hazards regression models. Curve fitting and Kaplan-Meier curves are visually illustrated. Statistical analyses were carried out using the R software package (http://www.R-project.org, The R Foundation), the nhanesR package, and the Free Statistics software version 1.8. Statistical significance was determined by a two-sided P value < 0.05.

## Results

### Demographics

The study analyzed a cohort of 1980 individuals with MetS, with 971 (49%) men and 1009 (51%) women with a mean age of 58 ± 16.5 years (Table 1). Participants were divided into three groups based on LTL, with Tertile 1 having the shortest LTL and Tertile 3 having the longest LTL. Significant differences were found between groups for age, Gender, weight, BMI, race, education, income, alcohol use, and smoking history. The shorter telomere group had older patients, more male participants, lower weight, a higher proportion of non-Hispanic whites, former smokers, and a higher percentage of heavy alcohol users. The longer telomere group was younger, had a higher proportion of females, higher BMI, a higher proportion of other Hispanics, never alcohol users, and current smokers.

**Table 1:** Baseline Characteristics of Participants with metabolic syndrome in NHANES 1999–2002

Characteristics	Total (n = 1980)	Tertile 1 (n = 660)	Tertile 2 (n = 660)	Tertile3 (n = 660)	P value
Age (years), Mean ± SD	58.0 ± 16.5	65.2 ± 14.0	57.6 ± 15.6	51.1 ± 16.6	< 0.001
Age group, n (%)					< 0.001
<45 years	434 (21.9)	60 (9.1)	142 (21.5)	232 (35.2)	
45–64 years	775 (39.1)	232 (35.2)	272 (41.2)	271 (41.1)	
≥ 65 years	771 (38.9)	368 (55.8)	246 (37.3)	157 (23.8)	
Gender, n (%)					0.001
Female	1009 (51.0)	307 (46.5)	329 (49.8)	373 (56.5)	
Male	971 (49.0)	353 (53.5)	331 (50.2)	287 (43.5)	
Height (cm), Mean ± SD	167.2 ± 10.3	166.8 ± 10.3	167.2 ± 10.5	167.5 ± 10.1	0.476
Weight(kg), Mean ± SD	86.8 ± 19.8	84.5 ± 19.0	88.0 ± 20.9	87.9 ± 19.2	0.001
BMI(kg/m^2^), Mean ± SD	31.0 ± 5.9	30.2 ± 5.5	31.4 ± 6.3	31.3 ± 5.9	< 0.001
Race, n (%)					0.002
Mexican American	438 (22.1)	151 (22.9)	162 (24.5)	125 (18.9)	
Non-Hispanic Black	335 (16.9)	90 (13.6)	110 (16.7)	135 (20.5)	
Non-Hispanic White	1075 (54.3)	384 (58.2)	350 (53)	341 (51.7)	
Other Hispanic	87 ( 4.4)	24 (3.6)	24 (3.6)	39 (5.9)	
Other Race - Including Multi-Racial	45 ( 2.3)	11 (1.7)	14 (2.1)	20 (3)	
Marital status, n (%)					< 0.001
Single	147 ( 7.4)	22 (3.3)	44 (6.7)	81 (12.3)	
Married	1249 (63.1)	421 (63.8)	427 (64.7)	401 (60.8)	
Divorced or separated or widowed	584 (29.5)	217 (32.9)	189 (28.6)	178 (27)	
Education, n (%)					< 0.001
College Graduate or above	351 (17.7)	103 (15.6)	124 (18.8)	124 (18.8)	
High School Grad/GED or Equivalent	475 (24.0)	149 (22.6)	152 (23)	174 (26.4)	
less than high school	691 (34.9)	277 (42)	216 (32.7)	198 (30)	
Some College or AA degree	463 (23.4)	131 (19.8)	168 (25.5)	164 (24.8)	
Alcohol user, n (%)					< 0.001
former	483 (24.4)	184 (27.9)	162 (24.5)	137 (20.8)	
heavy	324 (16.4)	74 (11.2)	103 (15.6)	147 (22.3)	
mild	646 (32.6)	222 (33.6)	228 (34.5)	196 (29.7)	
moderate	209 (10.6)	65 (9.8)	73 (11.1)	71 (10.8)	
never	318 (16.1)	115 (17.4)	94 (14.2)	109 (16.5)	
smoking, n (%)					< 0.001
former	704 (35.6)	266 (40.3)	249 (37.7)	189 (28.6)	
never	958 (48.4)	305 (46.2)	307 (46.5)	346 (52.4)	
now	318 (16.1)	89 (13.5)	104 (15.8)	125 (18.9)	
Annual family income, n (%)					< 0.001
$0 to $19,999	644 (32.5)	264 (40)	182 (27.6)	198 (30)	
$20,000 to $34,999	488 (24.6)	156 (23.6)	184 (27.9)	148 (22.4)	
$35,000 to $54,999	327 (16.5)	95 (14.4)	111 (16.8)	121 (18.3)	
more than $55,000	521 (26.3)	145 (22)	183 (27.7)	193 (29.2)	
Telomere Length (BP), Mean ± SD	5593.1 ± 591.4	5018.5 ± 202.4	5515.8 ± 131.2	6245.0 ± 479.6	< 0.001
Telomere T/S, Mean ± SD	1.0 ± 0.2	0.7 ± 0.1	0.9 ± 0.1	1.2 ± 0.2	< 0.001
Anemia, n (%)					0.956
Mild	90 ( 4.5)	31 (4.7)	30 (4.5)	29 (4.4)	
Moderate	30 ( 1.5)	11 (1.7)	11 (1.7)	8 (1.2)	
Non-Aemia	1859 (93.9)	618 (93.6)	619 (93.8)	622 (94.2)	
Severe	1 ( 0.1)	0 (0)	0 (0)	1 (0.2)	
Asthma, n (%)					0.579
no	1696 (85.7)	571 (86.5)	558 (84.5)	567 (85.9)	
yes	284 (14.3)	89 (13.5)	102 (15.5)	93 (14.1)	
Chronic kidney disease, n (%)					< 0.001
no	1463 (73.9)	435 (65.9)	491 (74.4)	537 (81.4)	
yes	517 (26.1)	225 (34.1)	169 (25.6)	123 (18.6)	
Angina, n (%)					0.291
no	1871 (94.5)	617 (93.5)	624 (94.5)	630 (95.5)	
yes	109 ( 5.5)	43 (6.5)	36 (5.5)	30 (4.5)	
Heart attack, n (%)					< 0.001
no	1868 (94.3)	603 (91.4)	631 (95.6)	634 (96.1)	
yes	112 ( 5.7)	57 (8.6)	29 (4.4)	26 (3.9)	
Congestive heart failure, n (%)					0.069
no	1895 (95.7)	623 (94.4)	632 (95.8)	640 (97)	
yes	85 ( 4.3)	37 (5.6)	28 (4.2)	20 (3)	
COPD, n (%)					0.008
no	1918 (96.9)	628 (95.2)	645 (97.7)	645 (97.7)	
yes	62 ( 3.1)	32 (4.8)	15 (2.3)	15 (2.3)	
Coronary heart disease, n (%)					0.331
no	1873 (94.6)	619 (93.8)	623 (94.4)	631 (95.6)	
yes	107 ( 5.4)	41 (6.2)	37 (5.6)	29 (4.4)	
DM, n (%)					0.515
DM	436 (23.1)	160 (24.7)	150 (23.7)	126 (20.9)	
IFG	205 (10.9)	73 (11.2)	69 (10.9)	63 (10.4)	
no	1244 (66.0)	416 (64.1)	414 (65.4)	414 (68.7)	
Hypertension, n (%)					< 0.001
no	601 (30.4)	165 (25)	201 (30.5)	235 (35.6)	
yes	1379 (69.6)	495 (75)	459 (69.5)	425 (64.4)	
Hyperlipidemia, n (%)					0.31
no	291 (14.7)	100 (15.2)	86 (13)	105 (15.9)	
yes	1689 (85.3)	560 (84.8)	574 (87)	555 (84.1)	
stroke, n (%)					0.073
no	1905 (96.2)	629 (95.3)	632 (95.8)	644 (97.6)	
yes	75 ( 3.8)	31 (4.7)	28 (4.2)	16 (2.4)	
Cancer, n (%)					0.007
no	1741 (87.9)	561 (85)	582 (88.2)	598 (90.6)	
yes	239 (12.1)	99 (15)	78 (11.8)	62 (9.4)	
All-cause mortality, n (%)					< 0.001
Alive	1161 (58.6)	281 (42.6)	415 (62.9)	465 (70.5)	
Dead	819 (41.4)	379 (57.4)	245 (37.1)	195 (29.5)	
Death cause, n (%)					0.105
Accidents	25 ( 3.1)	7 (1.8)	5 (2)	13 (6.7)	
Alzheimer’s disease	34 ( 4.2)	18 (4.7)	6 (2.4)	10 (5.1)	
Cerebrovascular diseases	44 ( 5.4)	24 (6.3)	13 (5.3)	7 (3.6)	
Chronic lower respiratory diseases	33 ( 4.0)	15 (4)	10 (4.1)	8 (4.1)	
Diabetes mellitus	33 ( 4.0)	11 (2.9)	10 (4.1)	12 (6.2)	
Cardiovascular disease	231 (28.2)	108 (28.5)	71 (29)	52 (26.7)	
Influenza and pneumonia	11 ( 1.3)	8 (2.1)	3 (1.2)	0 (0)	
Cancer	183 (22.3)	81 (21.4)	55 (22.4)	47 (24.1)	
Nephritis, nephrotic syndrome and nephrosis	12 ( 1.5)	7 (1.8)	2 (0.8)	3 (1.5)	
All other causes (residual)	213 (26.0)	100 (26.4)	70 (28.6)	43 (22.1)	
Follow time, Mean ± SD	15.3 ± 5.4	13.8 ± 6.1	15.6 ± 5.2	16.6 ± 4.3	< 0.001

### Mortality distribution

The study reported a total of 819 deaths including 231 cardiovascular deaths during the follow-up period. The distribution of deaths was not uniform among the tertiles, with the highest percentage of deaths occurring in the Tertile1 (57.4%), followed by the Tertile2 (37.1%), and the Tertile3 (29.5%). The causes of death were classified into different disease groups, with diseases of the heart being the leading cause of death, accounting for 28.2% of all deaths. Other causes of death included cancer (22.3%), cerebrovascular diseases (5.4%), chronic lower respiratory diseases (4.0%), diabetes mellitus (4.0%), Alzheimer’s disease (4.2%), nephritis/nephrotic syndrome/nephrosis (1.5%), influenza and pneumonia (1.3%), and accidental injuries (3.1%). While there was no statistically significant difference in the cause of death between the LTL tertiles, there was a lower percentage of deaths and the longest mean follow-up time in the Tertile1, suggesting a link between shorter telomere length and increased mortality risk in MetS patients.

### The relationship between LTL and all-cause mortality or CVD mortality

According to the fitted curves, shorter LTL in MetS increases all-cause mortality (Fig. 2) and CVD mortality (Fig. 3). Conversely, as telomere length increases, the risk of both all-cause and CVD mortality decreases significantly (Figs. 2 and 3). Kaplan-Meier survival curves indicated that low LTL was associated with an increased all-cause mortality and CVD mortality risk (*P < 0.05*) (Figs. 4 and 5).

**Fig. 1:**
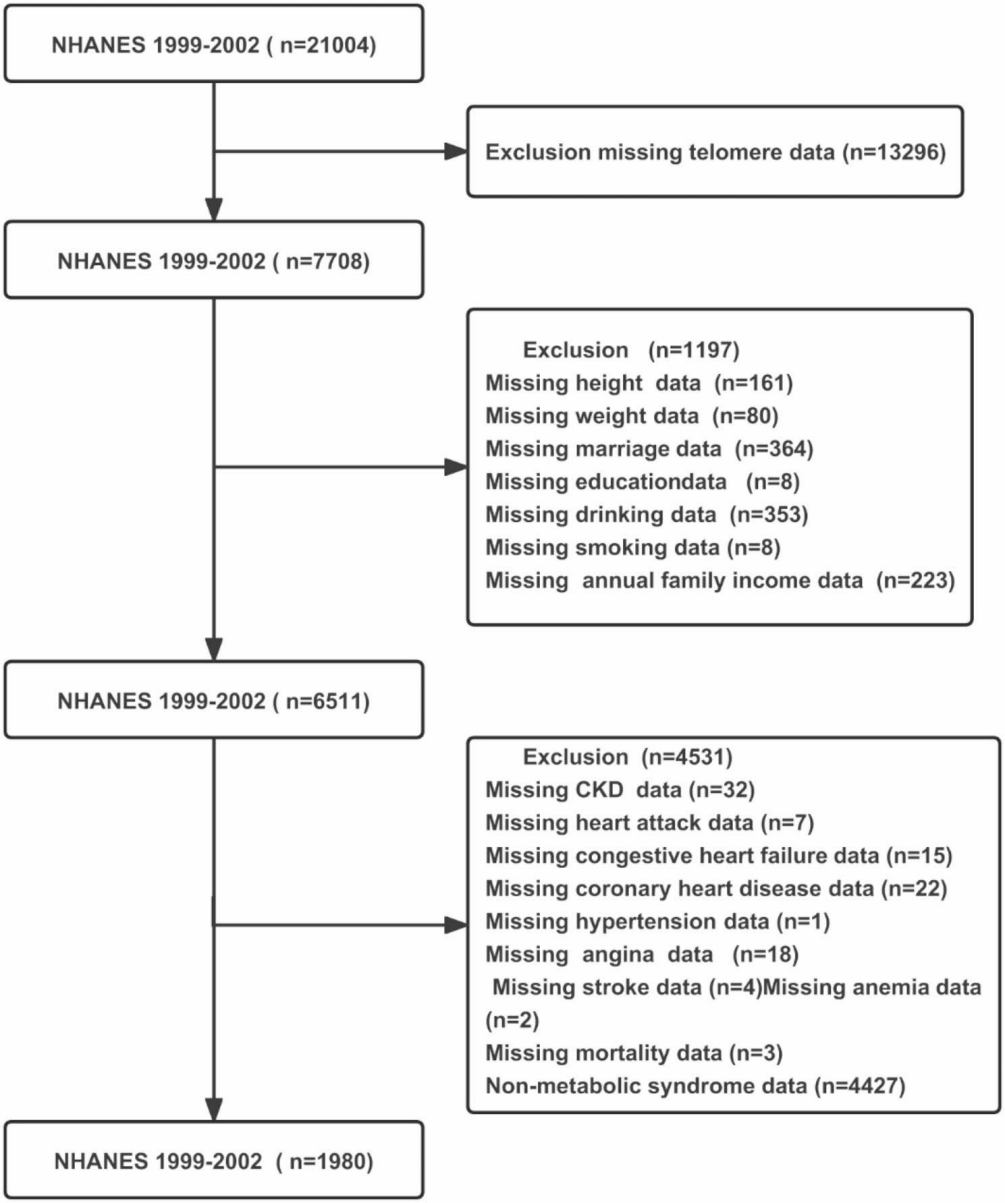
The flow chart of the participants with metabolic syndrome in NHANES of this study

**Fig. 2:**
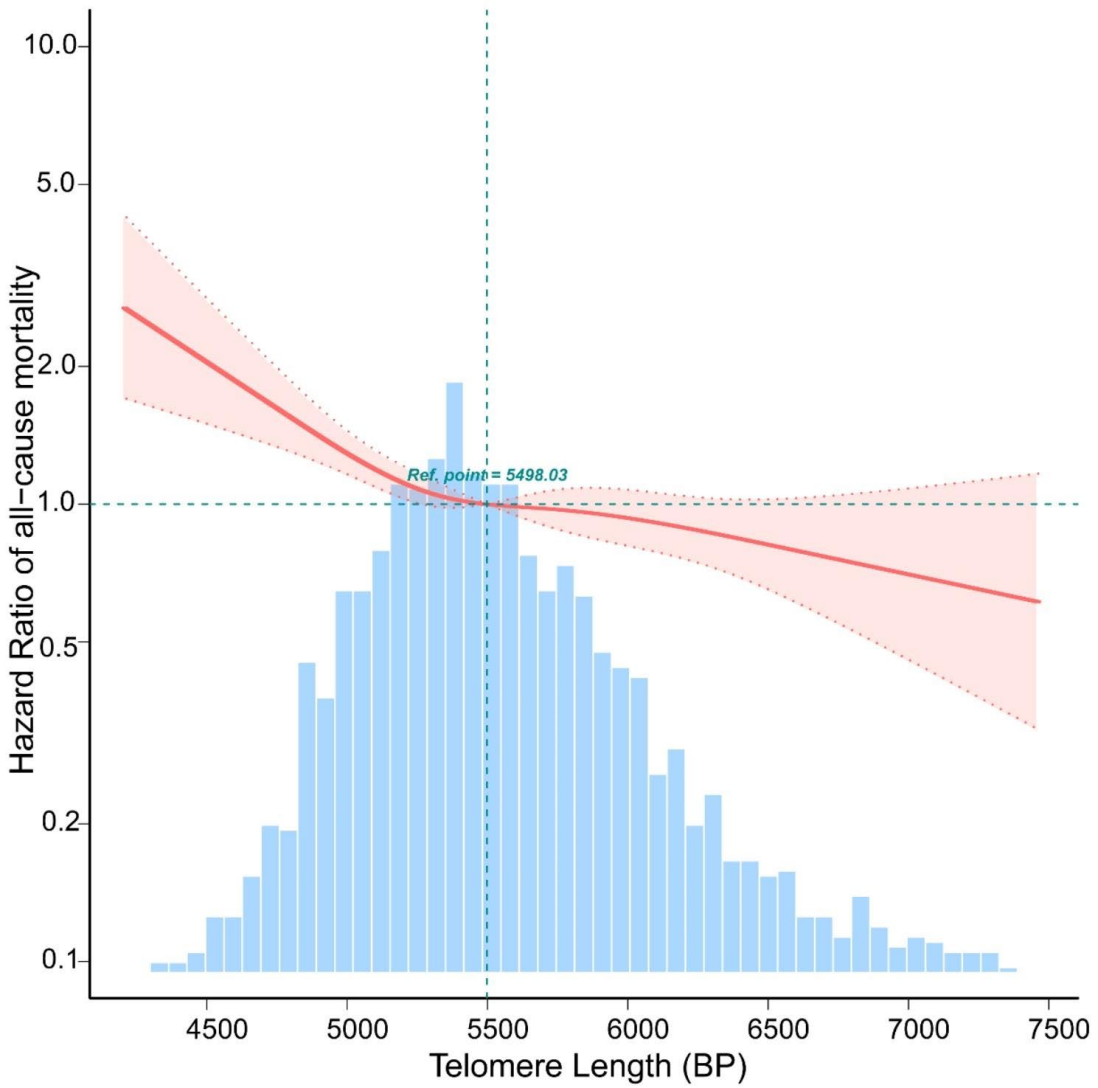
The relationship between Telomere Length with all-cause mortality by curve fitting in participants with metabolic syndrome

**Fig. 3:**
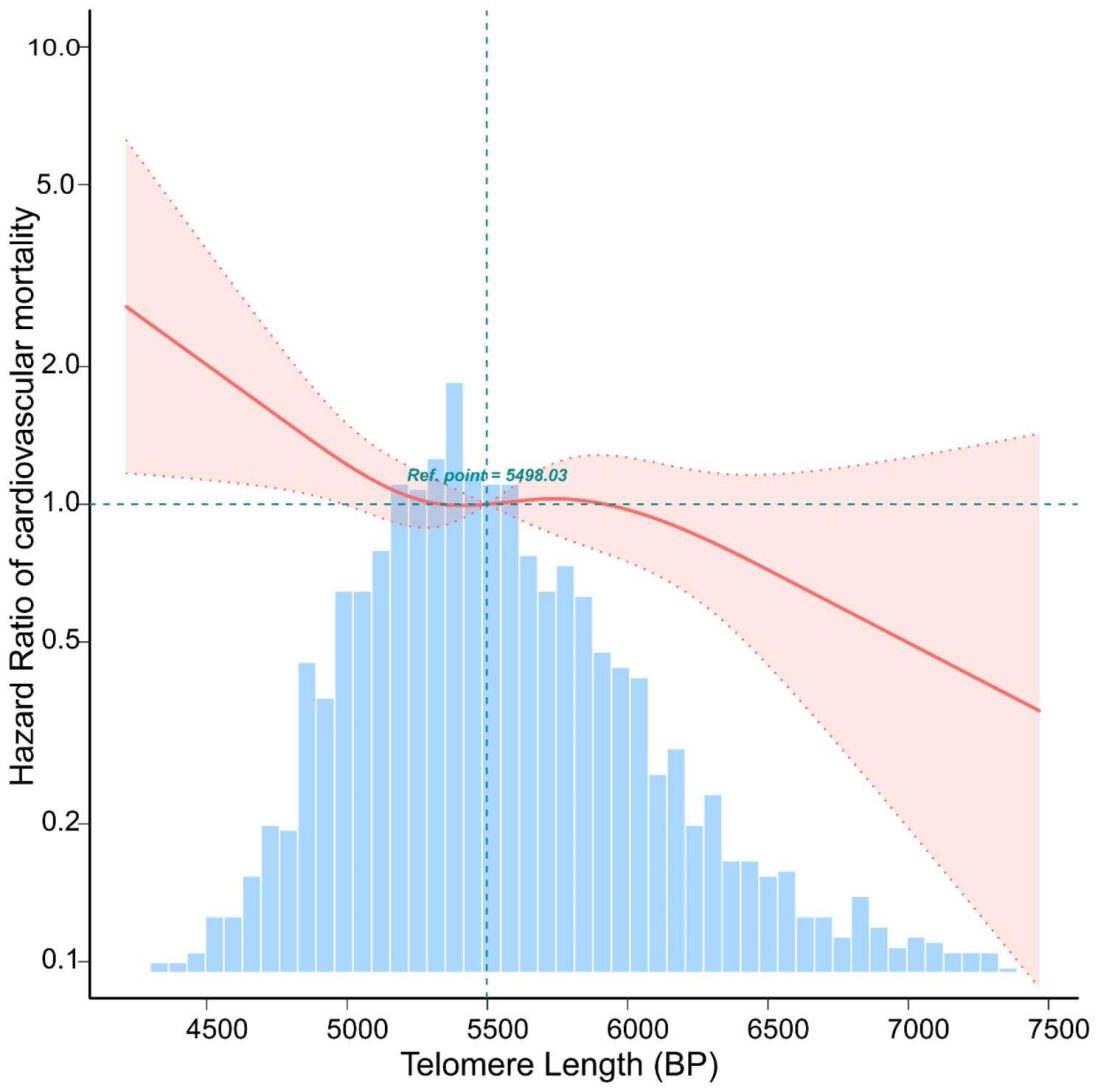
The relationship between Telomere Length with CVD mortality by curve fitting in participants with metabolic syndrome

**Fig. 4:**
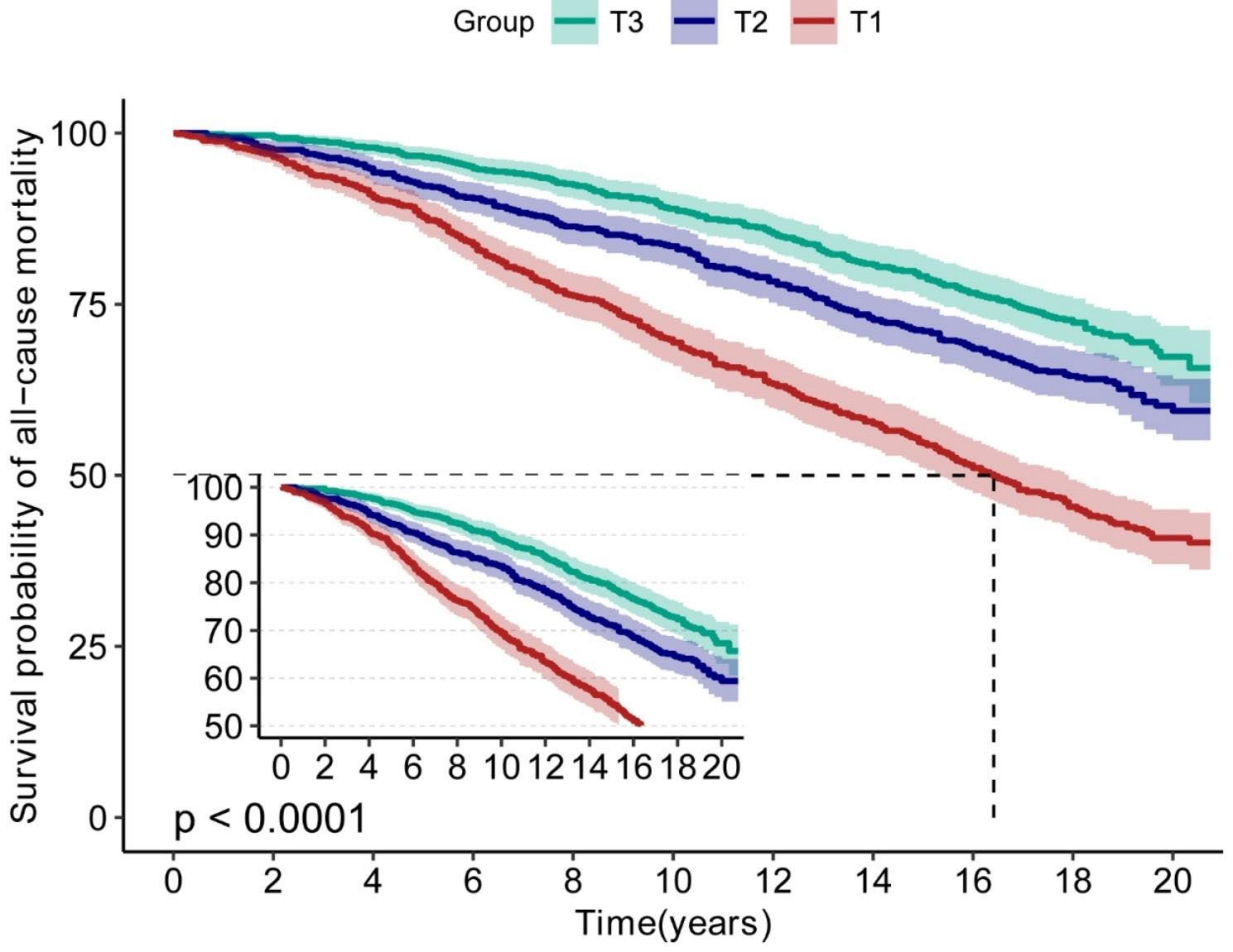
Kaplan-Meier survival curves for LTL associated with all-cause mortality risk

**Fig. 5:**
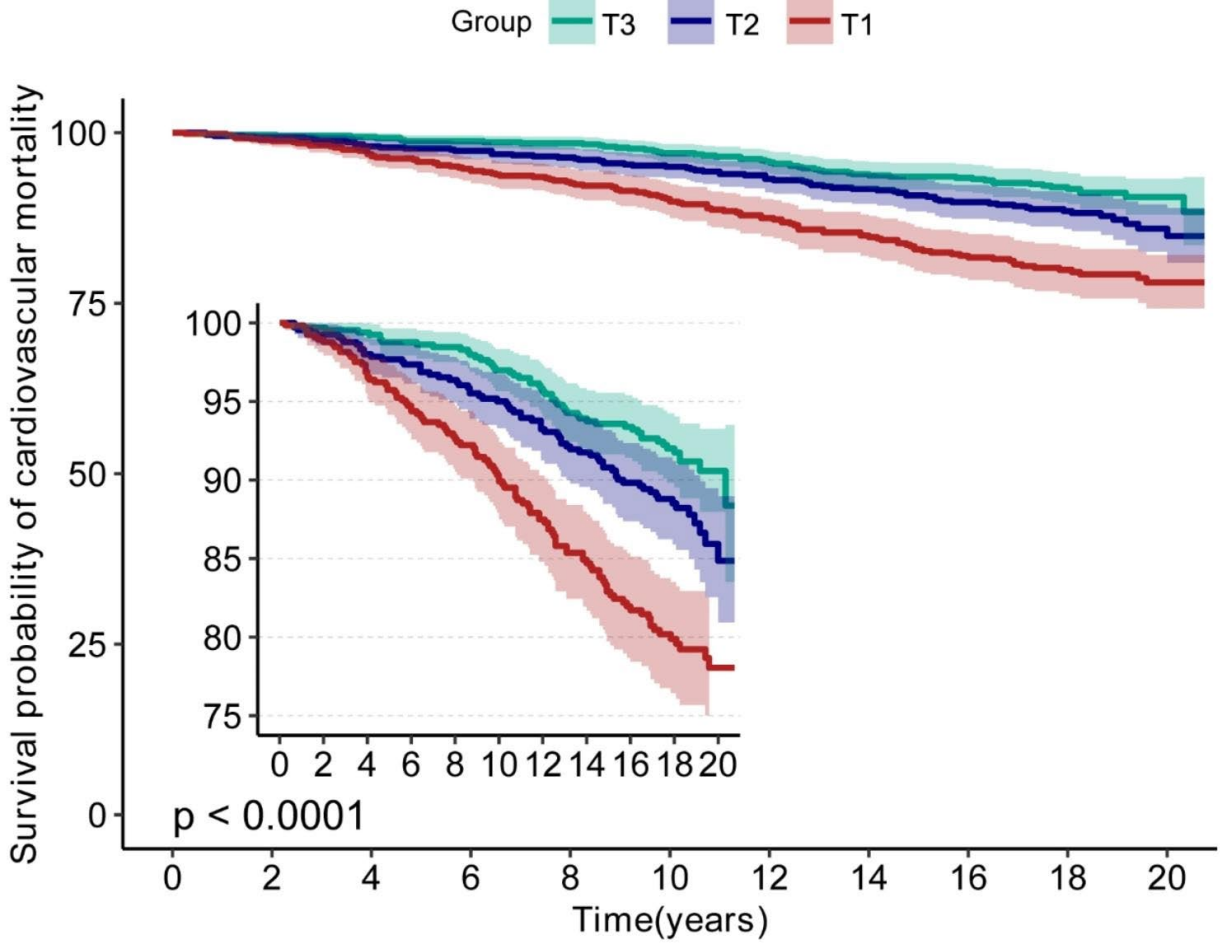
Kaplan-Meier survival curves for LTL associated with CVD mortality risk

Four models were used to stratify hazard ratios (HRs) for all-cause mortality and cardiovascular mortality by tertiles of telomere length (Table 2).

**Table 2:** The HRs of all-cause mortality or CVD mortality in the participants with metabolic syndrome

Variable	Total	Event(%)	Model 1	P value	Model 2	P value	Model 3	P value	Model 4	P value
All-cause mortality										
LTL Tertile3	660	195 (29.5)	1(Ref)		1(Ref)		1(Ref)		1(Ref)	
LTL Tertile2	660	245 (37.1)	1.35 (1.12 ~ 1.63)	0.002	1.03 (0.85 ~ 1.24)	0.788	1.03 (0.85 ~ 1.25)	0.769	1 (0.83 ~ 1.21)	0.999
LTL Tertile1	660	379 (57.4)	2.43 (2.05 ~ 2.89)	< 0.001	1.43 (1.19 ~ 1.7)	< 0.001	1.4 (1.17 ~ 1.68)	< 0.001	1.35 (1.12 ~ 1.61)	0.001
Trend test	1980	819 (41.4)	1.58 (1.45 ~ 1.73)	< 0.001	1.21 (1.11 ~ 1.33)	< 0.001	1.2 (1.1 ~ 1.32)	< 0.001	1.18 (1.08 ~ 1.29)	< 0.001
Cardiovascular mortality										
LTL Tertile3	660	52 (7.9)	1(Ref)		1(Ref)		1(Ref)		1(Ref)	
LTL Tertile2	660	71 (10.8)	1.47 (1.03 ~ 2.1)	0.036	1.09 (0.76 ~ 1.57)	0.626	1.11 (0.77 ~ 1.59)	0.589	1.1 (0.77 ~ 1.59)	0.594
LTL Tertile1	660	108 (16.4)	2.58 (1.85 ~ 3.59)	< 0.001	1.46 (1.04 ~ 2.04)	0.03	1.44 (1.02 ~ 2.03)	0.038	1.41 (1 ~ 2)	0.049
Trend test	1980	231 (11.7)	1.62 (1.38 ~ 1.92)	< 0.001	1.22 (1.03 ~ 1.45)	0.02	1.21 (1.02 ~ 1.44)	0.028	1.2 (1.01 ~ 1.42)	0.037

Model 1: Crude Model

Model 2: Adjusted for age and gender

Model 3: Adjusted for age, gender, race, marital status, body mass index, alcohol user, smoking status, and annual family income

Model 4: Adjusted for age, gender, race, marital status, body mass index, alcohol user, smoking status, annual family income, congestive heart failure, coronary heart disease, chronic kidney disease, chronic obstructive pulmonary disease (COPD), diabetes mellitus, hypertension, hyperlipidemia, and stroke

The crude model, which did not include any covariates, found that compared to individuals in Tertile 3, those in Tertile 2 and Tertile 1 had higher HRs for all-cause mortality: 1.35 (95% CI 1.12–1.63, P = 0.002) and 2.43 (95% CI 2.05–2.89, P < 0.001), respectively. A similar pattern was observed for cardiovascular mortality, with corresponding HRs of 1.47 (95% CI 1.03–2.10, P = 0.036) and 2.58 (95% CI 1.85–3.59, P < 0.001) for Tertile 2 and Tertile 1, respectively (Table 2).

The second model adjusted for age and gender as covariates. This analysis found that Tertile 1 had a significantly higher HR for all-cause mortality than Tertile 3 (1.43, 95% CI 1.19–1.70, P < 0.001), but no significant difference was observed between Tertile 2 and Tertile 3 (1.03, 95% CI 0.85–1.24, P = 0.788). For cardiovascular mortality, Tertile 1 had a higher HR than Tertile 3 (1.46, 95% CI 1.04–2.04, P = 0.030), whereas Tertile 2 did not (1.09, 95% CI 0.76–1.57, P = 0.626) (Table 2).

The third model was further adjusted for a variety of covariates, such as race, marital status, body mass index, alcohol use, smoking status, and annual family income. Tertile 1 remained significantly associated with a higher HR for all-cause mortality than Tertile 3 in this analysis (1.40, 95% CI 1.17–1.68, P0.001), whereas Tertile 2 did not (1.03, 95% CI 0.85–1.20, P = 0.769). For cardiovascular mortality, Tertile 1 had a higher HR than Tertile 3 (1.44, 95% CI 1.02–2.03, P = 0.038), while Tertile 2 did not (1.11, 95% CI 0.77–1.59, P = 0.589) (Table 2).

Finally, the fourth model included adjustment for a wide range of comorbidities in addition to the covariates included in Model 3, such as congestive heart failure, coronary heart disease, chronic kidney disease, COPD, diabetes mellitus, hypertension, hyperlipidemia, and stroke. This analysis found that Tertile 1 remained significantly associated with a higher HR for all-cause mortality (1.35, 95% CI 1.12–1.61, P = 0.001) compared to Tertile 3, while Tertile 2 did not (1.00, 95% CI 0.83–1.21, P = 0.999). Similarly, Tertile 1 had a higher HR than Tertile 3 for cardiovascular mortality (1.41, 95% CI 1.00–2.00, P = 0.049), while Tertile 2 did not (1.10, 95% CI 0.77–1.59, P = 0.594) (Table 2).

### Subgroup analyses

To investigate the robustness of the association between telomere length and all-cause mortality or cardiovascular (CVD) mortality in individuals with metabolic syndrome, stratified analyses were performed across various subgroups. None of the examined variables, including gender, chronic kidney disease, coronary heart disease, diabetes mellitus, hypertension, hyperlipidemia, stroke, and cancer, significantly modified the relationship between telomere length and the risk of all-cause mortality or CVD mortality in individuals with metabolic syndrome (all p-values for interaction > 0.05) (Table 3).

**Table 3:** The HRs of all-cause mortality or CVD mortality in the participants with metabolic syndrome in subgroup analyses

			All-cause mortality			Cardiovascular mortality	
Subgroup	Total	Event(%)	HR(95%CI)	P for interaction	Event(%)	HR(95%CI)	P for interaction
**Gender = Female**				0.726			0.115
LTL Tertile3	373	101 (27.1)	1(Ref)		19 (5.1)	1(Ref)	
LTL Tertile2	329	120 (36.5)	1.01 (0.77 ~ 1.33)		32 (9.7)	1.46 (0.81 ~ 2.62)	
LTL Tertile1	307	162 (52.8)	1.19 (0.92 ~ 1.55)		47 (15.3)	1.61 (0.92 ~ 2.81)	
Trend test	1009	383 (38)	1.1 (0.96 ~ 1.25)		98 (9.7)	1.24 (0.95 ~ 1.62)	
**Gender = Male**							
LTL Tertile3	287	94 (32.8)	1(Ref)		33 (11.5)	1(Ref)	
LTL Tertile2	331	125 (37.8)	0.91 (0.69 ~ 1.19)		39 (11.8)	0.82 (0.51 ~ 1.31)	
LTL Tertile1	353	217 (61.5)	1.16 (0.9 ~ 1.5)		61 (17.3)	0.91 (0.58 ~ 1.41)	
Trend test	971	436 (44.9)	1.1 (0.97 ~ 1.25)		133 (13.7)	0.97 (0.77 ~ 1.21)	
**Chronic kidney disease = no**				0.659			0.403
LTL Tertile3	537	128 (23.8)	1(Ref)		33 (6.1)	1(Ref)	
LTL Tertile2	491	145 (29.5)	0.92 (0.72 ~ 1.17)		37 (7.5)	0.86 (0.53 ~ 1.39)	
LTL Tertile1	435	197 (45.3)	1.15 (0.91 ~ 1.45)		54 (12.4)	1.09 (0.69 ~ 1.72)	
Trend test	1463	470 (32.1)	1.08 (0.96 ~ 1.22)		124 (8.5)	1.06 (0.84 ~ 1.34)	
**Chronic kidney disease = yes**							
LTL Tertile3	123	67 (54.5)	1(Ref)		19 (15.4)	1(Ref)	
LTL Tertile2	169	100 (59.2)	1.2 (0.87 ~ 1.65)		34 (20.1)	1.61 (0.89 ~ 2.91)	
LTL Tertile1	225	182 (80.9)	1.32 (0.98 ~ 1.79)		54 (24)	1.4 (0.8 ~ 2.44)	
Trend test	517	349 (67.5)	1.14 (0.99 ~ 1.32)		107 (20.7)	1.12 (0.87 ~ 1.45)	
**Corory heart disease = no**				0.351			0.695
LTL Tertile3	631	174 (27.6)	1(Ref)		43 (6.8)	1(Ref)	
LTL Tertile2	623	222 (35.6)	1 (0.82 ~ 1.22)		59 (9.5)	1.07 (0.71 ~ 1.59)	
LTL Tertile1	619	346 (55.9)	1.24 (1.02 ~ 1.49)		91 (14.7)	1.21 (0.83 ~ 1.77)	
Trend test	1873	742 (39.6)	1.12 (1.02 ~ 1.24)		193 (10.3)	1.11 (0.92 ~ 1.33)	
**Corory heart disease = yes**							
LTL Tertile3	29	21 (72.4)	1(Ref)		9 (31)	1(Ref)	
LTL Tertile2	37	23 (62.2)	0.69 (0.36 ~ 1.32)		12 (32.4)	0.85 (0.34 ~ 2.14)	
LTL Tertile1	41	33 (80.5)	1.12 (0.62 ~ 2.03)		17 (41.5)	1.39 (0.58 ~ 3.33)	
Trend test	107	77 (72)	1.07 (0.79 ~ 1.47)		38 (35.5)	1.21 (0.77 ~ 1.91)	
**DM = yes**				0.207			0.241
LTL Tertile3	126	66 (52.4)	1(Ref)		24 (19)	1(Ref)	
LTL Tertile2	150	74 (49.3)	0.87 (0.62 ~ 1.23)		21 (14)	0.71 (0.39 ~ 1.3)	
LTL Tertile1	160	113 (70.6)	1.02 (0.74 ~ 1.41)		37 (23.1)	0.98 (0.57 ~ 1.7)	
Trend test	436	253 (58)	1.02 (0.87 ~ 1.2)		82 (18.8)	1.01 (0.76 ~ 1.34)	
**DM = no**							
LTL Tertile3	477	126 (26.4)	1(Ref)		28 (5.9)	1(Ref)	
LTL Tertile2	483	169 (35)	0.98 (0.78 ~ 1.24)		50 (10.4)	1.23 (0.77 ~ 1.97)	
LTL Tertile1	489	266 (54.4)	1.25 (1 ~ 1.56)		71 (14.5)	1.33 (0.84 ~ 2.1)	
Trend test	1449	561 (38.7)	1.13 (1.02 ~ 1.27)		149 (10.3)	1.14 (0.92 ~ 1.42)	
**Hypertension = no**				0.846			0.689
LTL Tertile3	235	43 (18.3)	1(Ref)		10 (4.3)	1(Ref)	
LTL Tertile2	201	42 (20.9)	0.88 (0.57 ~ 1.37)		10 (5)	0.99 (0.4 ~ 2.46)	
LTL Tertile1	165	63 (38.2)	1.12 (0.75 ~ 1.7)		12 (7.3)	0.96 (0.39 ~ 2.37)	
Trend test	601	148 (24.6)	1.07 (0.87 ~ 1.32)		32 (5.3)	0.98 (0.63 ~ 1.54)	
**Hypertension = yes**							
LTL Tertile3	425	152 (35.8)	1(Ref)		42 (9.9)	1(Ref)	
LTL Tertile2	459	203 (44.2)	0.97 (0.78 ~ 1.2)		61 (13.3)	1.02 (0.68 ~ 1.52)	
LTL Tertile1	495	316 (63.8)	1.22 (1 ~ 1.5)		96 (19.4)	1.22 (0.84 ~ 1.78)	
Trend test	1379	671 (48.7)	1.12 (1.02 ~ 1.24)		199 (14.4)	1.12 (0.93 ~ 1.35)	
**Hyperlipidemia = no**				0.857			0.987
LTL Tertile3	105	37 (35.2)	1(Ref)		10 (9.5)	1(Ref)	
LTL Tertile2	86	34 (39.5)	0.83 (0.49 ~ 1.4)		10 (11.6)	1.06 (0.34 ~ 3.33)	
LTL Tertile1	100	59 (59)	1.24 (0.79 ~ 1.96)		15 (15)	1.18 (0.42 ~ 3.31)	
Trend test	291	130 (44.7)	1.15 (0.91 ~ 1.45)		35 (12)	1.09 (0.66 ~ 1.8)	
**Hyperlipidemia = yes**							
LTL Tertile3	555	158 (28.5)	1(Ref)		42 (7.6)	1(Ref)	
LTL Tertile2	574	211 (36.8)	0.97 (0.78 ~ 1.19)		61 (10.6)	1 (0.67 ~ 1.49)	
LTL Tertile1	560	320 (57.1)	1.17 (0.96 ~ 1.43)		93 (16.6)	1.16 (0.79 ~ 1.7)	
Trend test	1689	689 (40.8)	1.1 (0.99 ~ 1.21)		196 (11.6)	1.09 (0.9 ~ 1.31)	
**Stroke = no**				0.655			0.218
LTL Tertile3	644	183 (28.4)	1(Ref)		46 (7.1)	1(Ref)	
LTL Tertile2	632	226 (35.8)	0.97 (0.8 ~ 1.18)		67 (10.6)	1.11 (0.76 ~ 1.62)	
LTL Tertile1	629	351 (55.8)	1.18 (0.98 ~ 1.42)		100 (15.9)	1.21 (0.84 ~ 1.73)	
Trend test	1905	760 (39.9)	1.1 (1 ~ 1.21)		213 (11.2)	1.1 (0.92 ~ 1.31)	
**Stroke = yes**							
LTL Tertile3	16	12 (75)	1(Ref)		6 (37.5)	1(Ref)	
LTL Tertile2	28	19 (67.9)	0.91 (0.32 ~ 2.52)		4 (14.3)	0.41 (0.04 ~ 4.26)	
LTL Tertile1	31	28 (90.3)	1.74 (0.66 ~ 4.58)		8 (25.8)	1.33 (0.22 ~ 8.09)	
Trend test	75	59 (78.7)	1.46 (0.93 ~ 2.31)		18 (24)	1.32 (0.53 ~ 3.26)	
**Cancer = no**				0.425			0.138
LTL Tertile3	598	164 (27.4)	1(Ref)		44 (7.4)	1(Ref)	
LTL Tertile2	582	199 (34.2)	0.91 (0.74 ~ 1.13)		55 (9.5)	0.86 (0.57 ~ 1.29)	
LTL Tertile1	561	300 (53.5)	1.14 (0.93 ~ 1.39)		89 (15.9)	1.12 (0.77 ~ 1.64)	
Trend test	1741	663 (38.1)	1.08 (0.98 ~ 1.2)		188 (10.8)	1.09 (0.9 ~ 1.32)	
**Cancer = yes**							
LTL Tertile3	62	31 (50)	1(Ref)		8 (12.9)	1(Ref)	
LTL Tertile2	78	46 (59)	1.21 (0.75 ~ 1.97)		16 (20.5)	1.75 (0.69 ~ 4.42)	
LTL Tertile1	99	79 (79.8)	1.49 (0.95 ~ 2.33)		19 (19.2)	1.53 (0.62 ~ 3.78)	
Trend test	239	156 (65.3)	1.22 (0.98 ~ 1.52)		43 (18)	1.18 (0.77 ~ 1.81)	

Adjusted for age, Gender, race, marital status, body mass index, alcohol user, smoking status, and annual family income

## Discussion

Telomeres are repetitive DNA sequences located at the end of chromosomes. Their primary role is to protect the genetic material from being damaged or lost during cell division. Each time a cell divides, the telomeres shorten, and after multiple rounds of cell division, they become critically short and fragile [8]. Cellular aging and oxidative stress have both been linked to telomere shortening and implicated in the pathogenesis of MetS [8].

MetS is a crucial risk factor for mortality [21].MetS pathophysiology includes cellular aging and oxidative stress, both of which are associated with decreased TL. [22]。Some studies reveal that MetS patients have shortened telomeres, suggesting that telomere shortening may contribute to MetS development and progression [7, 9, 15]. Nellie Y. Loh et al. concluded through Mendelian randomization that longer leukocyte LTL is associated with an increased risk of upper body fat distribution, hypertension, and MetS [15]. The precise mechanism by which MetS patients have shortened LTL remains unclear, but it may involve chronic inflammation, insulin resistance, and mitochondrial dysfunction [8, 9, 23].

Numerous studies have shown that shortened telomere length increased the risk of all-cause mortality [10, 24, 25]。In a study involving 472,432 participants from the UK Biobank, Schneider et al. found that shortened LTL was positively correlated with all-cause mortality as well as disease-specific mortality for respiratory, digestive, circulatory, musculoskeletal, and infectious diseases [6]. Konstantin G Arbeev analyzed three European cohorts including a total of 3259 individuals and revealed that the risk of death from any cause increased by 1.34 times for every 1 kb decrease in LTL [10]. The Wilheit et al. study tracked 787 cancer patients for 15 years and showed a robust correlation between shorter telomeres and cancer death [24]. Likewise, Ayodeji Adegunsoye et al. found that leukocyte telomere length was a biomarker for predicting mortality among 2046 patients with pulmonary fibrosis [25]。 Our study confirms this relationship and extends it to patients with metabolic syndrome. We discovered that in individuals with MetS, shorter LTL is associated with an increased risk of death, even after taking into account a variety of demographic and clinical factors. The median follow-up time in our study was 17.75 years, which is relatively long compared to other studies examining LTL and mortality rates, pointing to the importance of LTL as a biomarker for detecting high-risk populations in clinical practice.

Telomere shortening is significantly associated with cardiovascular disease (CVD), heart failure, and disease-specific mortality rates [23, 26–29]. Metabolic syndrome is also a high-risk group for CVD. Age, inflammation, obesity, sedentary lifestyle, smoking, psychological stress, and oxidative stress are all risk factors for CVD that are also linked to telomere shortening [30]. Data from 2,151,597 middle-aged people showed that metabolic syndrome is a risk factor for heart failure in middle-aged men and women, with a higher impact on women [2]. In a cohort study of 290 patients with acute myocardial infarction, LTL was identified as a significant predictor of all-cause mortality and CVD mortality one year after myocardial infarction [31]. Genetic evidence has also identified several variants of telomere maintenance genes that may increase the risk of heart disease [30]. Telomere shortening can accelerate cellular aging and impair function, fueling atherosclerosis and other vascular diseases [30]. Studies indicate that weight loss surgery and calorie restriction can decrease BMI and increase telomere length in MetS patients, providing insights for managing their health [32]. However, a few studies indicate that short telomere burden and average LTL are not robust independent predictors of atherosclerosis at the preclinical stage [33]. Our findings emphasize the link between shortened telomere length and an elevated risk of CVD mortality in people with MetS. Shortened telomeres may serve as a potential biomarker and therapeutic target for CVD [30].

A possible explanation for the observed association is that shorter LTL is a marker of unhealthy aging. Our study found that individuals with shorter LTL were older, more likely to be male, have lower weight, and have a higher percentage of heavy alcohol use, suggesting that LTL could serve as a predictor of unhealthy aging in MetS patients. The process of cellular senescence is another hypothesized mechanism linking shorter LTL with higher mortality risk. When telomeres get too short, cells go through a process called senescence, which involves the release of pro-inflammatory chemicals and the permanent halting of cell division [34, 35]. Cellular senescence is thought to contribute to various age-related pathologies, including osteoarthritis, atherosclerosis, and frailty [22, 36, 37]. Thus, the link between shorter LTL and mortality in MetS patients could, in part, be mediated by the induction of cellular senescence.

The mechanism by which telomere shortening affects mortality risk is complex and likely involves multiple pathways and processes [6]. One potential mechanism is cellular senescence, a process that leads to the permanent cessation of cell division in response to telomere attrition [34]. Senescent cells can contribute to tissue dysfunction and inflammation, which have been associated with increased mortality risk in MetS patients. Additionally, telomere shortening can lead to oxidative stress, which can cause DNA damage, inflammation, and cellular dysfunction, which have all been linked to increased mortality risk in MetS patients [35]. Telomere shortening can also promote chronic inflammation and activation of the immune system, which has been implicated in a variety of age-related diseases, including MetS and its associated complications [34].

Our study possesses several strengths, including a large sample size and comprehensive adjustment for multiple confounding factors. Furthermore, our study had a long follow-up period, with a median follow-up time of 17.75 years, enabling us to evaluate the enduring mortality risk associated with LTL and MetS. However, it is important to acknowledge that our study was observational in design, thus precluding the establishment of causality. An important limitation of our study is the assessment of telomere length at a single time point, which may not capture potential fluctuations in telomere length over time. Additionally, the study population was drawn from the United States, and the results may not be generalizable to other populations with different demographics. Future research is warranted to investigate how changes in telomere length over time can impact the mortality risk in individuals with MetS.

Our analysis confirms that MetS patients with shorter LTL have higher all-cause and CVD mortality. LTL may be a biomarker for high-risk people who could benefit from focused interventions to reduce the risk of death. To create effective treatments for preventing age-related diseases, further research is needed to better understand the mechanisms behind the association between telomere length, MetS, and mortality risk.

## Data Availability

Publicly available datasets were analyzed in this study. This data can be found at: https://www.cdc.gov/nchs/nhanes/index.htm.
